# Influence of Pistachios on Performance and Exercise-Induced Inflammation, Oxidative Stress, Immune Dysfunction, and Metabolite Shifts in Cyclists: A Randomized, Crossover Trial

**DOI:** 10.1371/journal.pone.0113725

**Published:** 2014-11-19

**Authors:** David C. Nieman, Johannes Scherr, Beibei Luo, Mary Pat Meaney, Didier Dréau, Wei Sha, Dustin A. Dew, Dru A. Henson, Kirk L. Pappan

**Affiliations:** 1 Appalachian State University, Human Performance Lab, North Carolina Research Campus, Kannapolis, NC, United States of America; 2 Department of Prevention and Sports Medicine, Klinikum rechts der Isar, Technische Universitaet Muenchen, Munich, Germany; 3 Department of Physiology, Second Military Medical University, 800 Xiangyin Road, Shanghai, 200433, China; 4 Charlotte Research Institute & Department of Biological Sciences, University of North Carolina, Charlotte, NC, United States of America; 5 Bioinformatics Services Division, University of North Carolina at Charlotte, North Carolina Research Campus, Kannapolis, NC, United States of America; 6 Department of Biology, Immunology Laboratory, Appalachian State University, Boone, NC, United States of America; 7 Metabolon Inc., Durham, NC, United States of America; Vanderbilt University, United States of America

## Abstract

**Objectives:**

Pistachio nut ingestion (3 oz./d, two weeks) was tested for effects on exercise performance and 21-h post-exercise recovery from inflammation, oxidative stress, immune dysfunction, and metabolite shifts.

**Methods:**

Using a randomized, crossover approach, cyclists (N = 19) engaged in two 75-km time trials after 2-weeks pistachio or no pistachio supplementation, with a 2-week washout period. Subjects came to the lab in an overnight fasted state, and ingested water only or 3 oz. pistachios with water before and during exercise. Blood samples were collected 45 min pre-exercise, and immediately post-, 1.5-h post-, and 21-h post-exercise, and analyzed for plasma cytokines, C-reactive protein (CRP), F_2_-isoprostanes (F_2_-IsoP), granulocyte phagocytosis (GPHAG) and oxidative burst activity (GOBA), and shifts in metabolites.

**Results:**

Performance time for the 75-km time trial was 4.8% slower under pistachio conditions (2.84±0.11 and 2.71±0.07 h, respectively, P = 0.034). Significant time effects were shown for plasma cytokines, CRP, F_2_-IsoP, GPHAG, and GOBA, with few group differences. Metabolomics analysis revealed 423 detectable compounds of known identity, with significant interaction effects for 19 metabolites, especially raffinose, (12Z)-9,10-Dihydroxyoctadec-12-enoate (9,10-DiHOME), and sucrose. Dietary intake of raffinose was 2.19±0.15 and 0.35±0.08 mg/d during the pistachio and no pistachio periods, and metabolomics revealed that colon raffinose and sucrose translocated to the circulation during exercise due to increased gut permeability. The post-exercise increase in plasma raffinose correlated significantly with 9,10-DiHOME and other oxidative stress metabolites.

**Conclusions:**

In summary, 2-weeks pistachio nut ingestion was associated with reduced 75-km cycling time trial performance and increased post-exercise plasma levels of raffinose, sucrose, and metabolites related to leukotoxic effects and oxidative stress.

**Trial Registration:**

ClinicalTrials.gov NCT01821820

## Introduction

Prolonged and intensive exercise causes transient physiologic stress, elevations in biomarkers related to inflammation and oxidative stress, immune dysfunction, and disturbances in host pathogen defense [1]. Immunonutrition support for athletes is a burgeoning area of scientific investigation, and a variety of nutritional products have been tested as countermeasures to exercise-induced indicators of physiologic stress [2]. Carbohydrate ingestion before, during, and after heavy exercise has emerged as an effective but partial countermeasure to immune dysfunction, with favorable effects on measures related to stress hormones and inflammation, but limited effects on oxidative stress and markers of innate or adaptive immunity. Other products that have been tested with mixed utility include vitamins and minerals, glutamine and other amino acids, β-glucan, flavonoids and polyphenols, N-3 polyunsaturated fatty acids (N-3 PUFAs or fish oil), unique plant extracts, and food products (e.g., chocolate milk, bananas, and raisins) [3].

In a randomized, crossover study with 14 trained cyclists, we showed that acute ingestion of bananas (with water) or a commercial sports drink supported 75-km cycling performance and underlying metabolic processes (as measured with metabolomics) to a similar degree when the rate of carbohydrate delivery was equated [4]. Exercise-induced inflammation, oxidative stress, and changes in innate immune function were also comparable between banana and sports drink trials, and at attenuated levels during recovery when compared to previous water-only trials that we have conducted [5]. There is growing interest in using water with food products instead of sports drinks to improve nutrient intake and health while supporting performance and recovery.

Pistachios (480 kcal per 3 oz serving) are nutrient-dense nuts that contain a unique nutrient profile of proteins and carbohydrates (∼30% of energy), fats (∼70% of energy), minerals (in particular, copper, iron, magnesium), potassium, vitamins B6 and thiamin, carotenoids, phytosterols, and phenolic acids [6]. Among tree nuts, pistachios are one of the best sources for water- and fat-soluble antioxidants. The nutrient mix in pistachio nuts has been associated with reductions in oxidative stress and inflammation in community trials and thus may serve as an effective countermeasure to exercise-induced inflammation and oxidative stress, but this has not yet been investigated [7], [8].

We hypothesized that ingestion of 3 ounces (85 g) of pistachio nuts per day for two weeks before and the day of cycling intensely for 75 kilometers would support performance and substrate utilization compared to water only, and attenuate inflammation, oxidative stress, and immune dysfunction during 21 hours of recovery. Metabolomics is the simultaneous measurement of all detectable small molecules or metabolites present in biological samples such as biofluids, tissues, and cellular extracts to elucidate the effect of a particular stimulus on metabolic pathways [9]. The use of metabolomics in exercise and nutritional sciences is gaining momentum [10], and global metabolomics profiling was utilized in this study to help capture the potential influence of pistachio nut supplementation in countering physiologic stress indicators associated with intense and prolonged exercise.

## Materials and Methods

The protocol for this trial and supporting CONSORT checklist are available as Protocol S1 and Checklist S1.

### Subjects

Subjects included 20 male cyclists (ages 27–49 years) who regularly competed in road races (category 1 to 5) and were capable of cycling 75-km at race pace. During the 6-week study, subjects maintained their typical training regimen (192±18 km cycling per week), maintained weight, and avoided the use of large-dose vitamin and mineral supplements, herbs, and medications. Subjects signed informed consent and study procedures were approved by the Institutional Review Board at Appalachian State University (ASU). The study was conducted during April to June, 2013, at the ASU Human Performance Laboratory at the North Carolina Research Campus in Kannapolis, NC.

### Research Design

**Figure 1:**
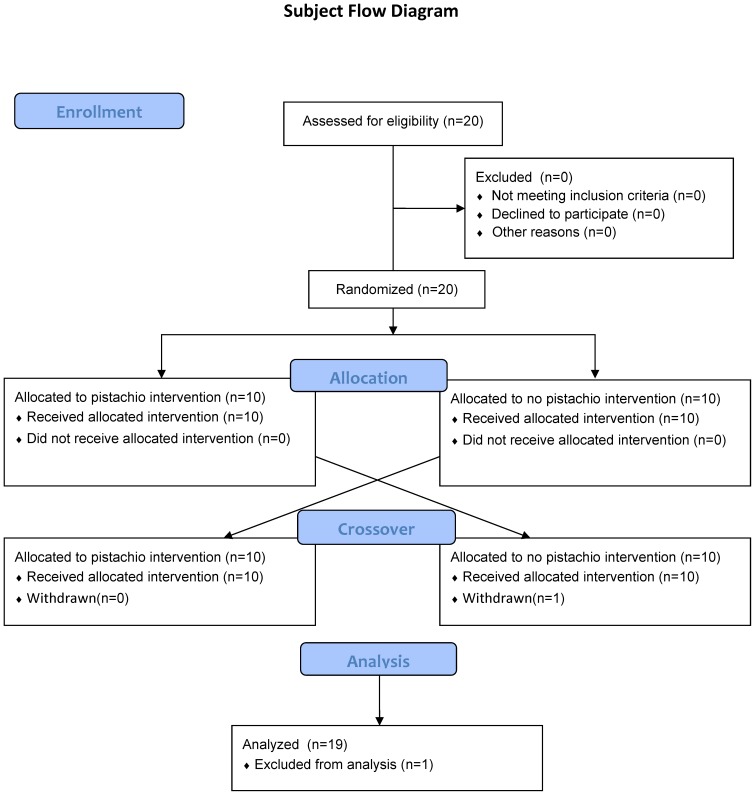
Subject flow diagram.

This study utilized a randomized (1∶1 allocation, random number generator), crossover approach, and subjects engaged in two 75-km cycling time trials after two weeks of pistachio or no pistachio supplementation (no blinding), with a 2-week washout period (Figure 1). Subjects completed both arms of the study, and data were analyzed with subjects operating as their own controls. Data were analyzed from subjects (N = 19) completing all aspects of the study using a repeated measures ANOVA, within subjects approach. One subject was unable to complete the second 75-km cycling time trial due to a schedule conflict. A full nutrient profile of the 3 oz. (85 g) daily serving size of pistachio nuts used in this study is available in Table S1.

Two weeks prior to the first 75-km time trial, athletes completed orientation and baseline testing. Demographic and training histories were acquired with questionnaires. A blood sample was collected. Maximal power, oxygen consumption, ventilation, and heart rate were measured during a graded exercise test (25 Watts increase every two minutes, starting at 150 Watts) with the Cosmed Quark CPET metabolic cart (Rome, Italy) and the Lode cycle ergometer (Lode Excaliber Sport, Lode B.V., Groningen, Netherlands). Body composition was measured with the Bod Pod body composition analyzer (Life Measurement, Concord, CA). Half of the subjects (randomized) left the lab with a 2-weeks supply of pistachio nuts (3 oz/day). Compliance to the pistachio nut supplementation regimen was assessed through weekly contact with the subjects via email, and by collecting empty plastic bags (one per day) from the subjects when reporting to the lab.

During the 3-day period prior to each 75-km cycling trial, subjects were asked to reduce the volume of their exercise training as if preparing for a race, and ingested a moderate-carbohydrate diet using a food list restricting high fat foods, other nuts and visible fats. Subjects recorded all food and beverage intake during the 3-day period, with macro- and micro-nutrient intake assessed using the Food Processor dietary analysis software system (ESHA Research, Salem, OR).

Two weeks following baseline testing, subjects returned to the Human Performance Laboratory at 6:45 am in an overnight fasted state (no food or beverages other than water for at least 9 hours), and provided a pre-exercise blood sample. Subjects either consumed 1.5 oz. pistachio nuts with water or water alone at 7:00 am, and rested 30 minutes. At 7:30 am, subjects warmed up and then began the 75-km cycling time trial using their own bicycles on CompuTrainer Pro Model 8001 trainers (RacerMate, Seattle, WA). A mountainous 75-km course with moderate difficulty was utilized using the CompuTrainer software system. Heart rate and rating of perceived exertion (RPE) were recorded every 30 minutes, and workload continuously monitored using the CompuTrainer MultiRider software system (version 3.0, RacerMate, Seattle, WA). Oxygen consumption and ventilation were measured using the Cosmed Quark CPET metabolic cart after 16 km and 55 km cycling. All subjects consumed 3 ml/kg water every 15 min, with subjects randomized to the pistachio condition consuming 1.5 oz pistachios after 1 h cycling. No other beverage or food containing energy or nutrients were allowed during the cycling time trials. Blood samples were taken via venipuncture immediately after completing the 75-km time trial, and then 1.5-h post-exercise. Subjects returned in an overnight fasted state the next morning to provide a 21-h post-exercise blood sample. Subjects washed out for two weeks, returned to the lab to provide a blood sample, crossed over to the opposite condition, and repeated all procedures.

### Complete Blood Count, Lactate, Glucose

Complete blood counts (CBC) were performed using a Coulter Ac.TTM 5Diff Hematology Analyzer (Beckman Coulter, Inc., Miami, FL). Shifts in plasma volume due to exercise were calculated using the equation of Dill and Costill [11]. Blood glucose and lactate were measured using microfuge tubes lined with EDTA dipotassium salt (RAM Scientific Inc., Germany), and the YSI 2300 STAT Plus Glucose and Lactate analyzer (Yellow Springs, OH).

### F_2_-isoprostanes

Plasma F_2_-isoprostanes (F_2_-IsoP) were determined using gas chromatography mass spectrometry (GC-MS) [12]. Plasma was collected from heparinized blood, immediately flash-frozen in liquid nitrogen, and stored at −80°C. Immediately prior to assay plasma samples were thawed, and free F_2_-IsoP extracted with deuterated [^2^H_4_] prostaglandin F_2_α added as an internal standard. The mixture was then added to a C18 SepPak column, followed by silica solid phase extractions. F_2_-IsoP were converted to pentafluorobenzyl esters, subjected to thin layer chromatography, and converted to trimethylsilyl ether derivatives. Samples were analyzed by a negative ion chemical ionization GC-MS using an Agilent 6890N gas chromatography interfaced to an Agilent 5975B inert MSD mass spectrometer (Agilent Technologies, Inc. Santa Clara, CA).

### C-Reaction Protein and Cytokines

High-sensitivity C-reactive protein (CRP) was measured using an LX-20 clinical analyzer (Beckman Coulter Electronics, Brea, CA). Total plasma concentrations of six inflammatory cytokines [monocyte chemoattractant protein-1 (MCP-1), tumor necrosis factor alpha (TNFα), granulocyte colony-stimulating factor (GCSF), IL-6, IL-8, and IL-10] were determined using an electrochemiluminescence based solid-phase sandwich immunoassay (Meso Scale Discovery, Gaithersburg,MD, USA). All samples and provided standards were analyzed in duplicate, and the intra-assay CV ranged from 1.7% to 7.5% and the inter-assay CV 2.4 to 9.6% for all cytokines measured. Pre-and post-exercise samples for the cytokines were analyzed on the same assay plate to decrease inter-kit assay variability.

### Granulocyte Phagocytosis (GPHAG) and Oxidative Burst Activity (GOBA)

GPHAG was measured through the uptake of Fluorescein isothiocyanate (FITC)-labeled *Staphylococcus aureus* bacteria and GOBA was measured through the oxidation of non-fluorescent hydroethidine (HE) to fluorescent ethidium bromide in cells stimulated with unlabeled bacteria. Samples were processed on a Q-Prep Workstation (Beckman Coulter, Inc.) and analysis was performed using a Beckman Coulter FC10 500LSR Fortessa flow cytometer (BD Biosciences, San Jose, CA). After gating on the granulocyte population using forward scatter and side scatter (FlowJo analysis Software, Tree Star Inc., Ashland, OR), the mean fluorescence intensity (MFI; x-mean) and percent positive cells for FITC (FL1) and oxidized HE (FL2) were determined.

### Metabolomics

The non-targeted metabolic profiling instrumentation employed for this analysis combined three independent platforms: ultrahigh performance liquid chromatography/tandem mass spectrometry (UHPLC/MS/MS) optimized for basic species, UHPLC/MS/MS optimized for acidic species, and gas chromatography/mass spectrometry (GC/MS) [13], [14]. Blood samples were collected in EDTA tubes and centrifuged at 3,000 RPM for 10 minutes at 4°C, with the plasma aliquoted, snap frozen in liquid nitrogen, and then stored at −80°C until analysis. For each plasma sample, 100 µL was used for analyses. Using an automated liquid handler (Hamilton LabStar, Salt Lake City, UT), protein was precipitated from the plasma with methanol that contained four standards to report on extraction efficiency. The resulting supernatant was split into equal aliquots for analysis on the three platforms. Aliquots, dried under nitrogen and vacuum-desiccated, were subsequently either reconstituted in 50 µL 0.1% formic acid in water (acidic conditions) or in 50 µL 6.5 mM ammonium bicarbonate in water, pH 8 (basic conditions) for the two UHPLC/MS/MS analyses or derivatized to a final volume of 50 µL for GC/MS analysis using equal parts bistrimethyl-silyl-trifluoroacetamide and solvent mixture acetonitrile:dichloromethane: cyclohexane (5∶4∶1) with 5% triethylamine at 60°C for 1 hour. In addition, three types of controls were analyzed in concert with the experimental samples: aliquots of a well-characterized human plasma pool served as technical replicates throughout the data set, extracted water samples served as process blanks, and a cocktail of standards spiked into every analyzed sample allowed instrument performance monitoring. Standards to monitor extraction were d6-cholesterol, fluorophenylglycine, and tridecanoic acid. A standard to monitor GC/MS derivatization was 2-tert butyl-6-methylphenol (BHT). GC/MS standards to monitor GC and MS performance were C5–C18 alkylbenzenes. Experimental samples and controls were randomized across platform run days.

For UHLC/MS/MS analysis, aliquots were separated using a Waters Acquity UPLC (Waters, Millford, MA) instrument with separate acid/base-dedicated 2.1 mm × 100 mm Waters BEH C18 1.7 µm particle columns heated to 40°C and analyzed using an LTQ mass spectrometer (Thermo Fisher Scientific, Inc., Waltham, MA) which consisted of an electrospray ionization (ESI) source and linear ion-trap (LIT) mass analyzer [14]. Extracts reconstituted in formic acid were gradient eluted at 350 µL/min using (A) 0.1% formic acid in water and (B) 0.1% formic acid in methanol (0% B to 70% B in 4 min, 70–98% B in 0.5 min, 98% B for 0.9 min), whereas extracts reconstituted in ammonium bicarbonate used (A) 6.5 mM ammonium bicarbonate in water, pH 8, and (B) 6.5 mM ammonium bicarbonate in 95/5 methanol/water (same gradient profile as above) at 350 µL/min. The MS instrument scanned 99–1000 m/z and alternated between MS and MS2 scans using dynamic exclusion with approximately 6 scans per second. Derivatized samples for GC/MS were separated on a 5% diphenyl/95% dimethyl polysiloxane fused silica column with helium as the carrier gas and a temperature ramp from 60°C to 340°C and then analyzed on a Thermo-Finnigan Trace DSQ MS (Thermo Fisher Scientific, Inc.) operated at unit mass resolving power with electron impact ionization and a 50–750 atomic mass unit scan range. Metabolites were identified by automated comparison of the ion features in the experimental samples to a reference library of chemical standard entries that included retention time, molecular weight (m/z), preferred adducts, and in-source fragments as well as associated MS spectra, and were curated by visual inspection for quality control using software developed at Metabolon Inc. (Durham, NC) [15]. Common and biologically abundant isomers of unsaturated fatty acids containing the n3, n6, and n9 configuration are contained in Metabolon's chemical standard library. LC/MS standards to monitor LC and MS performance were d_3_-leucine, chloro- and bromo-phenylalanine, d_2_-maleic acid, amitriptyline, and d10-benzophenone (1). Biochemical identifications were based on three criteria: retention index within a narrow window of the proposed identification, accurate mass match to the library +/- 0.005 amu, and the MS/MS forward and reverse scores between the experimental data and authentic standards. The MS/MS scores were based on a comparison of the ions present in the experimental spectrum to the ions present in the library spectrum.

### Statistical Analysis

Data are expressed as mean ± SE. Food record and performance data were compared between conditions (pistachio, water) using paired t-tests. Biomarker data were analyzed using a 2 (condition) x 5 (time) repeated-measures ANOVA, within-subjects design, with changes over time within conditions contrasted between conditions using paired t-tests and significance adjusted to P<0.0125 after Bonferroni correction. For the metabolomics statistical analyses and data display purposes, any missing values were assumed to be below the limits of detection and these values were imputed with the compound minimum (minimum value imputation). Statistical analysis of log-transformed data was performed using “R” (http://cran.r-project.org/), which is a freely available, open-source software package. Two-way repeated ANOVA with post-hoc contrasts (t-tests) was performed to compare data between conditions. An estimate of the false discovery rate (Q-value) was calculated to take into account the multiple comparisons that normally occur in metabolomics-based studies, with Q<0.05 used as an indication of high confidence in a result. Fold changes across time points were calculated using group averages of the median scaled intensity values. Intensity differences between pistachio and no-pistachio trials were calculated at each time point for each metabolite and protein biomarker. Pearson's product-moment correlation coefficients was used to determine if the changes of intensity difference over time were correlated between metabolites or proteins, with FDR adjustment conducted to account for multiple tests and Q≤0.05 used as an indication of significance.

## Results

The analysis included 19 of 20 competitive male cyclists (ages 27 to 49 years) who successfully adhered to all aspects of the study design (see Table 1). The aim of this study was to find outcome measures that might differ between trials with medium to large effects. At an effect size of 0.7 and alpha of 0.05, N = 19 provided a power of 0.823. The participants in this study were well-trained cyclists who exercised intensively on a regular basis. Therefore, the 75-km cycling time trials utilized in this study were unlikely to result in significant learning or training effects. Paired t-tests were used to compare pre-trial outcome measures for the pistachio and no-pistachio arms of the study, and no differences were found.

**Table 1:** Subject characteristics (N = 19).

Variable	Mean±SE
Age (years)	38.0±1.6
Height (m)	1.81±0.02
Weight (kg)	76.8±2.3
Body fat (%)	14.0±1.0
Watts_max_	304±10.5
VO_2max_^ (ml.kg.−1min−1)^	51.7±1.4
HR_max_ (beats/min)	179±2.5
Training (km/wk)	192±18.0

Three-day food record nutrient data are summarized in Table 2. Energy intake was 35% higher during the pistachio compared to no-pistachio supplementation period, with significantly higher intake measured for total fat, carbohydrate, and protein intake. Total fiber intake was 63% higher during pistachio supplementation, with 3-fold and 5.3-fold higher levels measured for insoluble fiber and raffinose [16], respectively. Pistachio supplementation was also associated with higher intake of a variety of vitamins and minerals including vitamin B6, folate, potassium, iron, copper, magnesium, zinc, and molybdenum.

**Table 2:** Daily averages from 3-day food record data (mean±SE).

Variable	Pistachio	Water	P-Value
**Energy (kJ)**	12.82±1.07	9.46±0.67	0.001
**Protein (g)**	121.3±12.1	89.1±7.5	0.005
**Carbohydrate (g)**	355±37.1	296±26.2	0.021
**Fiber (g)**	35.7±3.8	21.9±2.7	<0.001
**Insoluble fiber (g)**	13.2±0.9	3.30±0.57	<0.001
**Soluble fiber (g)**	2.20±0.52	1.03±0.20	0.027
**Raffinose (g)**	2.19±0.15	0.35±0.08	<0.001
**Total fat (g)**	130±10.2	73.0±7.3	<0.001
**Saturated fat (g)**	33.5±3.4	23.5±3.0	0.005
**Polyunsaturated fat (g)**	22.7±1.8	9.43±1.2	<0.001
**Monounsaturated fat (g)**	44.9±3.3	18.0±2.3	<0.001
**Cholesterol (mg)**	277±31.0	276±38.4	0.984
**Vitamin A (IU)**	7197±2045	4054±694	0.120
**Vitamin C (mg)**	102±11.0	74.0±9.6	0.040
**Vitamin E (mg αTE)**	12.4±3.5	5.20±0.73	0.048
**Vitamin D (IU)**	129±26.1	119±18.6	0.447
**Thiamin (mg)**	2.55±0.26	1.96±0.46	0.325
**Vitamin B6 (mg)**	3.43±0.42	1.74±0.15	0.002
**Folate (µg)**	499±63.2	301±29.8	0.004
**Potassium (mg)**	3479±325	2091±192	0.001
**Calcium (mg)**	1082±144	920±69.3	0.247
**Iron (mg)**	26.4±2.9	18.6±2.1	0.009
**Copper (mg)**	2.47±0.31	0.94±0.10	<0.001
**Magnesium (mg)**	373±42.0	236±29.3	0.004
**Zinc (mg)**	13.4±1.7	8.68±0.91	0.017
**Molybdenum (µg)**	41.3±4.7	14.5±2.9	<0.001
**Alcohol (g)**	13.7±2.0	18.0±3.0	0.324
**Caffeine (mg)**	129±22.1	146±20.6	0.433

**Figure 2:**
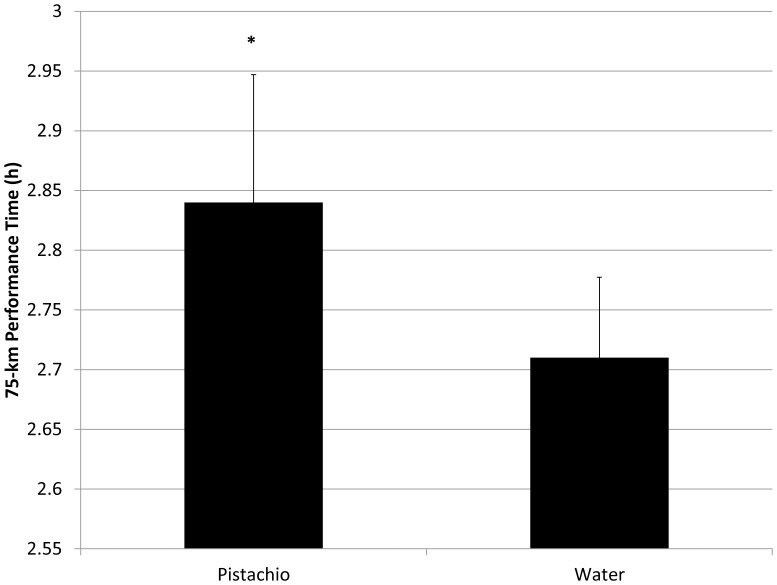
Performance time for the 75-km trial under pistachio compared to water-only conditions (mean±SE). *  =  Q-value 2-way ANOVA contrasts <0.05.

Performance data from the 75-km mountainous cycling time trial are summarized in Table 3 and Figure 2. Performance time for the 75-km trial was 4.8% slower under pistachio compared to water-only conditions (2.84±0.11 and 2.71±0.07 h, respectively, P = 0.034) (Figure 2). Oxygen consumption (P = 0.014) and ventilation (P = 0.038) were lower in the pistachio versus water trial, with a trend towards reduced power output (P = 0.114). Blood lactate increases were similar between pistachio and water trials, with a trend for better maintenance of blood glucose levels during the pistachio trial (interaction effect, P = 0.064). Changes in plasma volume and body weight were similar between pistachio and water trials.

**Table 3:** Metabolic and performance data during the 75-km cycling trials under pistachio and water conditions in trained cyclists (N = 19) (mean±SE).

Variable	Pistachio	Water	P-Value
**VO_2_ (ml.kg.−1min−1)**	34.1±1.2	35.6±1.1	0.014
**VO_2_ (%VO_2max_)**	66.6±2.6	69.3±2.1	0.210
**Watts**	201±8.5	207±7.6	0.114
**% Watts_max_**	66.6±2.6	68.3±1.7	0.262
**HR (beats/min)**	149±3.6	150±2.5	0.562
**%HR_max_**	83.0±1.7	84.0±1.1	0.309
**Ventilation (L/min)**	71.0±4.0	74.9±3.5	0.038
**RPE**	13.2±0.2	12.9±0.3	0.334
**RER**	0.88±0.01	0.89±0.01	0.375
**Plasma volume shift (%)**	−9.62±0.92	−9.37±1.64	0.904
**Blood lactate (mmol/L)**			**Interaction**
Pre-Exercise	0.59±0.03	0.65±0.05	P = 0.226
Post-Exercise	1.95±0.30	1.67±0.11	
**Blood glucose (mmol/L)**			
Pre-Exercise	4.16±0.12	4.25±0.12	P = 0.064
Post-Exercise	3.90±0.21	3.48±0.21	
**Body Weight (kg)**			
Pre-Exercise	77.0±2.3	76.7±2.1	P = 0.165
Post-Exercise	76.0±4.9	75.3±2.1	

VO_2_, volume of oxygen consumed; HR, heart rate; RPE, rating of perceived exertion; RER, respiratory exchange ratio (VCO_2_/VO_2_).

Plasma cytokine, CRP, F_2_-Isop, total blood leukocyte, and granulocyte phagocytosis and oxidative burst activity data are summarized in Table 4. Significant time effects were measured for all of the variables in Table 4, with no differences in the patterns of change assessed between pistachio and water trials except for CRP (interaction effect, P = 0.037) and F_2_-IsoP (P = 0.035). Two-way ANOVA contrasts indicated slightly lower CRP and higher F_2_-IsoP in the pistachio compared to water trial at 21-h post-exercise, but P-values were not significant after Bonferroni correction.

**Table 4:** Comparison between pistachio and water trials for inflammation, oxidative, and immune biomarkers in trained cyclists (N = 19) (mean±SE).

Variable	Pre-Suppl	Pre-Exercise	Post-Exercise	1.5-h Post-Exercise	21-h Post-Exercise	P-values: Time; Interaction
**IL-6 (pg/ml)**						
**Pistachio**	0.70±0.06	0.88±0.16	9.37±1.67	5.79±0.92	1.25±0.39	<0.001
**Water**	0.68±0.05	1.02±0.28	8.77±0.69	5.97±0.97	0.63±0.05	0.521
**IL-8 (pg/ml)**						
**Pistachio**	5.04±0.37	5.84±0.49	13.7±1.5	10.7±1.1	5.88±0.80	<0.001
**Water**	4.81±0.39	5.23±0.42	11.8±1.1	7.91±0.74	4.70±0.33	0.420
**IL-10 (pg/ml)**						
**Pistachio**	2.14±0.25	2.21±0.21	38.3±13.7	16.7±4.3	2.39±0.21	0.008
**Water**	2.08±0.20	2.00±0.19	33.7±10.8	16.9±4.7	2.12±0.24	0.861
**TNFα (pg/ml)**						
**Pistachio**	3.73±0.19	3.86±0.18	4.59±0.26	4.19±0.23	3.76±0.18	<0.001
**Water**	3.67±0.16	3.90±0.16	4.30±0.20	4.10±0.20	3.62±0.16	0.143
**MCP-1 (pg/ml)**						
**Pistachio**	249±9.3	271±9.5	373±19.6	330±16.0	245±12.7	<0.001
**Water**	261±11.6	267±9.1	385±18.9	340±22.5	230±7.1	0.355
**GCSF (pg/ml)**						
**Pistachio**	12.1±0.7	11.6±0.8	16.5±1.3	18.4±1.5	15.6±1.7	0.001
**Water**	11.1±0.8	12.8±1.0	17.7±1.8	18.7±2.2	15.3±1.5	0.431
**CRP (mg/L)**						
**Pistachio**	1.12±0.28	1.81±0.36	1.92±0.40	1.75±0.36	3.77±0.47	0.001
**Water**	1.22±0.30	1.60±0.36	1.71±0.40	1.68±0.39	5.39±0.82	0.037
**F_2_Isop (pg/ml)**						
**Pistachio**	33.0±2.6	33.9±2.1	57.3±3.8	52.6±4.5	40.8±3.8	<0.001
**Water**	38.4±3.1	45.4±4.7	62.5±5.4	53.6±3.5	36.4±3.1	0.035
**Leukocytes (10^9^/L)**						
**Pistachio**	5.38±0.24	5.33±0.25	16.4±0.9	12.7±0.6	5.41±0.34	<0.001
**Water**	5.21±0.25	5.51±0.28	16.4±0.9	12.7±0.7	5.63±0.23	0.900
**GPHAG (MFI)**						
**Pistachio**	7133±478	7948±477	12098±625	10615±763	7079±455	<0.001
**Water**	8122±526	8156±564	11761±480	10733±546	7491±458	0.319
**GOBA (MFI)**						
**Pistachio**	1028±36.3	1120±45.5	858±31.0	858±34.4	896±80.4	<0.001
**Water**	1059±27.4	1072±31.4	902±27.1	875±34.7	802±98.1	0.604

IL  =  interleukin; TNFα = tumor necrosis factor alpha; MCP-1 = monocyte chemoattractant protein-1; GCSF = granulocyte colony-stimulating factor; CRP = C-reactive protein; F_2_Isop = F_2_-isoprostanes; GPHAG = granulocyte phagocytosis; GOBA = granulocyte oxidative burst activity; MFI = mean fluorescence intensity.

**Figure 3:**
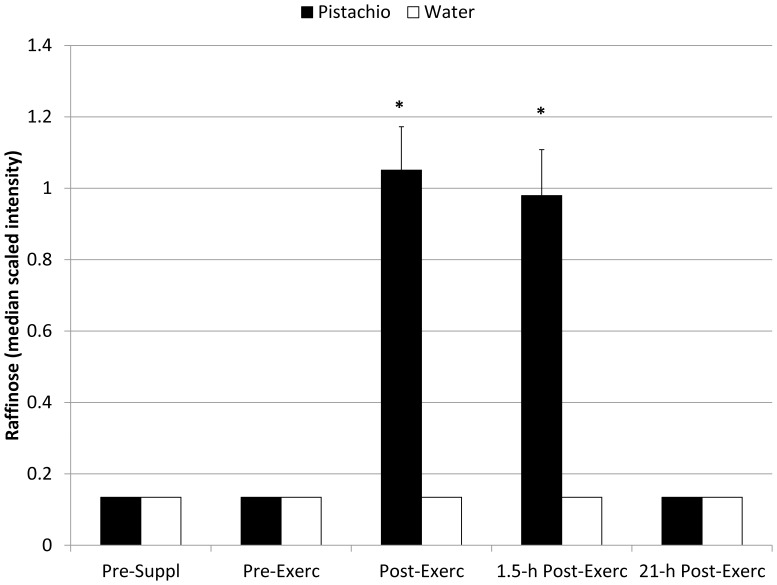
Plasma raffinose in pistachio and water conditions (interaction effect, Q<0.001) (mean±SE). *  =  Q-value 2-way ANOVA contrasts <0.05.

**Figure 4:**
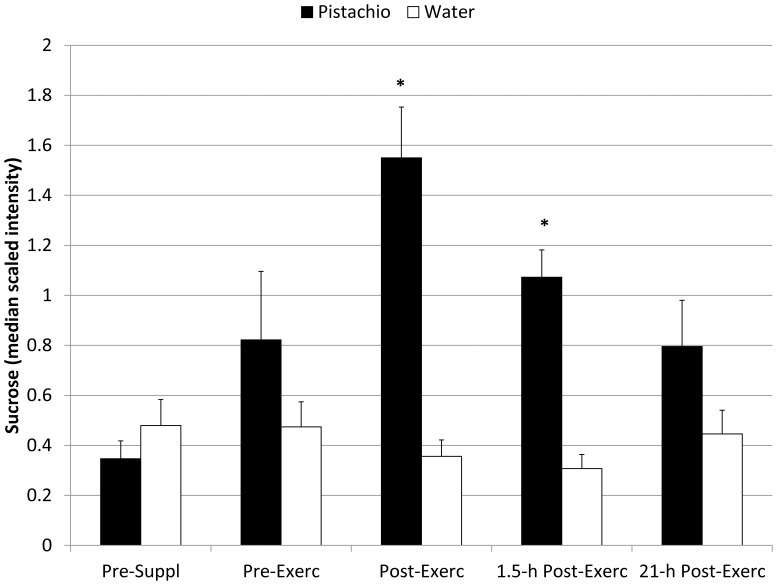
Plasma sucrose in pistachio and water conditions (interaction effect, Q<0.001) (mean±SE). *  =  Q-value 2-way ANOVA contrasts <0.05.

**Figure 5:**
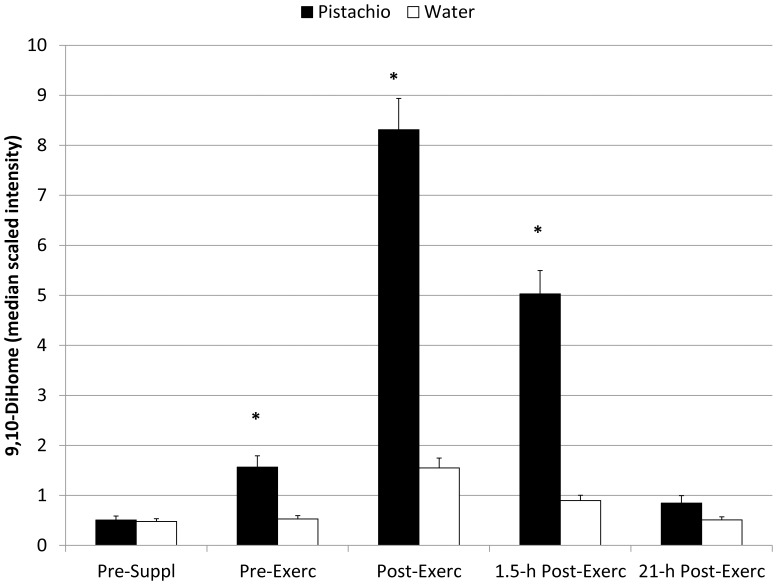
Plasma 9,10 DiHOME in pistachio and water conditions (interaction effect, Q<0.001) (mean±SE). *  =  Q-value 2-way ANOVA contrasts <0.05.

The metabolomics analysis revealed 423 detectable compounds of known identity with 2-way ANOVA contrasts indicating post-exercise increases for 167 metabolites (Table S2). Following log transformation and imputation with minimum observed values for each compound, 2×5 repeated measures ANOVA interaction effects were significant for the 19 metabolites listed in Table 5. File S1 contains Figures S1 to S19 depicting the box plots for these 19 metabolites. Fold differences were calculated from the median scaled intensity values for each of the five time points and then rank ordered according to post-exercise values. The greatest fold differences post-exercise were measured for raffinose, sucrose, and (Z)-9,10-dihydroxyoctadec-12-enoic acid (9,10 DiHOME), and the pattern of change over time for these three metabolites during the pistachio and water trials are compared in Figures 3, 4, and 5. Other metabolites that were increased to higher levels post-exercise in the pistachio compared to water trials included two secondary bile acids (glycodeoxycholate, glycochenodeoxycholate), a product of uric acid oxidation (allantoin), amino acids and products of amino acid metabolism (leucine, indoleacetate, 2-aminooctanoate, gamma-glutamylleucine, gamma-glutamyltyrosine, phenylacetate, isobutyrylcarnitine), products of fatty acid metabolism (2-hydroxydecanoate, azelate), two lysolipids (1- and 2-oleoylglycerophosphoethanolamine), and a food component of pistachios (*myo*-inositol). Pre-to-post-exercise changes of the intensity difference (between pistachio and no-pistachio trials) in raffinose were strongly correlated with sucrose (r = 0.88, Q = 0.048), 9,10-DiHOME (r = 0.95, Q = 0.033), *myo*-inositol (r = 0.99, Q = 0.027), allantoin (r = 0.97, Q = 0.030), and azelate (r = 0.96, Q = 0.031), but not with CRP (r = 0.45, Q = 0.311), IL-6 (r = 0.10, Q = 0.825), or F_2_-isoprostanes (r = 0.08, Q = 0.860).

**Table 5:** Metabolites with significant treatment (pistachio, water) x time (5 points) interaction effects.

Metabolite	Pre-Suppl	Pre-Exerc	Post-Exerc	1.5-h Post-Exerc	21-h Post-Exerc	Interaction q-Value
**raffinose**	1	1	7.81*	7.28*	1	<0.001
**9,10-DiHOME**	1.06	2.96*	5.36*	5.6*	1.66	<0.001
**sucrose**	0.72	1.73	4.35*	3.49*	1.78	<0.001
**glycodeoxycholate**	0.87	1.61	4.31*	1.96*	2.18	0.006
**2-hydroxydecanoate**	1.21	2.58*	3.21*	3.17*	2.27*	<0.001
**glycochenodeoxycholate**	0.8	1.38	2.68*	1.64*	1.45	0.024
**allantoin**	1.36	1.13	2.55*	2.08*	1.23	0.016
**phenylacetate**	0.72	1.74	2.24*	1.8	1.37	0.032
**phenylacetylglutamine**	0.98	1.64*	2.05*	2.07*	1.53*	0.023
**1-oleoylglycerophos-phoethanolamine**	1.08	1.01	1.7*	1.62*	0.92	<0.001
**2-oleoylglycerophos-phoethanolamine**	1.19	1.05	1.69*	1.61*	0.89	0.042
**azelate**	0.97	0.99	1.64*	1.43*	1.07	<0.001
**isobutyrylcarnitine**	0.98	1.27	1.63*	1.43*	1.22	0.024
**indoleacetate**	1.04	1.56*	1.61*	1.53*	1.29	0.015
**2-aminooctanoate**	1.02	1.55*	1.58*	1.62*	1.45*	0.006
**myo-inositol**	1.09	1.02	1.4*	1.45*	0.99	0.015
**gamma-glutamylleucine**	1.03	1.08	1.31*	1.25*	1.07	0.015
**gamma-glutamyltyrosine**	0.96	1.05	1.2*	1.26*	0.98	0.023
**leucine**	0.99	1.14*	1.18*	1.14*	1.04	0.014

Values represent the ratio of pistachio-to-water median scaled intensity values at each time point (mean±SE).

* = Q-value 2-way ANOVA contrasts <0.05.

## Discussion

Contrary to our hypothesis, data from this randomized, crossover study showed that 2-weeks ingestion of pistachio nuts (3 oz./day including the day of the cycling time trial) by trained cyclists impaired performance 4.8% during a 75-km cycling time trial. Although the patterns of change for traditional biomarkers for exercise-induced inflammation and oxidative stress were similar between pistachio and no-pistachio trials, metabolomics revealed trial differences for 19 metabolites highlighted by the post-exercise presence of raffinose, sucrose, *myo*-inositol, and the leukotoxin diol 9,10-DiHOME in the blood compartment of athletes during the pistachio trial.

Pistachio nuts contain the trisaccharide raffinose, a soluble carbohydrate also found in onions and many types of legumes [16]. Raffinose family oligosaccharides have multiple functions in plants, serving as transport and storage carbohydrates, and cryoprotectants in frost-hardy plant organs [17]. The biosynthesis of raffinose is linked to primary plant metabolism via *myo*-inositol, sucrose, and galactose [17], [18]. Raffinose is poorly absorbed in the human small intestine which lacks α-galactosidases, but is metabolized by colon bacteria to sucrose, melibiose, fructose, galactose, and glucose [19]. Raffinose is an effective prebiotic, stimulating short-chain fatty acid production and the growth of beneficial colon bacteria such as bifidobacteria and lactobacilli that have been related to positive effects on bowel function, inflammatory bowel disease, cancer risk, and intestinal epithelial and dendritic cell function [20].

Pistachio nuts also contain phytates in a concentration that varies from 0.29 to 2.83 g/100 g [21]. Phytic acid (inositol hexakisphosphate or phytate when in salt form) is the principal storage form of phosphorus in many plant tissues, especially bran, legumes, seeds, and nuts. Most phytate hydrolysis occurs in the large intestine by means of microbial phytases, resulting in *myo*-inositol and other inositol phosphates [21].

The athletes in this study ingested 3 oz./day shelled pistachios for the 2-week period before the 75-km cycling time trial, and then ingested a total of 3 oz. pistachios before and during exercise after arriving in the lab in an overnight fasted state. The trial differences seen in this study for post-exercise metabolite shifts were more than likely due to chronic pistachio ingestion because of the multiple hours needed for transit time to the colon for undigested components. Prolonged and intensive exercise is associated with hyperthermia, splanchnic hypoperfusion, loss of intestinal barrier integrity, and increased intestinal permeability or the so-called ‘leaky gut’ syndrome [22]–[24]. Although not previously reported, the significant post-exercise increase in plasma raffinose, sucrose, and *myo*-inositol in athletes during the pistachio arm of the study suggests that these biochemicals translocated from the colon due to exercise-induced increases in gut permeability. Plasma sucrose is widely used as a marker of increased gastrointestinal permeability [22]. The athletes came to the lab having ingested high levels of pistachios for two weeks, and their colons more than likely contained substantial pistachio raffinose and sucrose (from bacterial degradation of raffinose) and *myo*-inositol (from bacterial phytase hydrolysis of phytate). Post-exercise increases in plasma levels of two secondary bile acids were also measured during the pistachio trial, suggesting that pistachio raffinose and phytates may have amplified exercise-induced increases in gut permeability.

We previously reported in another metabolomics-based study that supplementation with a blueberry and green tea polyphenol-rich soy protein-based product for 17 days caused a distinct gut-derived phenolic signature in long distance runners following a 3-day period of intensified running [25]. Metabolomics is ideally suited as a methodology to investigate shifts in gut-derived metabolites following supplementation with dietary supplements or whole foods, and human trials are revealing an increasing number of metabolites that appear at high levels in the colon and systemic circulation [26]. The biological relevance for most of these gut-derived metabolites that increase transiently in athletes following intensive exercise bouts is still being explored. The dominant phenolics in pistachios are gallic acid, protocatechuic acid, catechins and epicatechins, rutin, eriodictyol, quercetin, luteolin, myricetin, and cyanidin-3-galactoside [27]. However, we were unable to detect a gut-derived phenolic signature when comparing metabolite shifts following 75-km cycling under pistachio and no-pistachio conditions, and this was more than likely related to the relatively modest phenolic intake in this study compared to the study with the blueberry and green tea polyphenol-rich soy protein-based product [25].

The transient increase in plasma 9,10-DiHOME following exercise in the pistachio trial was strongly correlated with increases in plasma raffinose. 9,10-DiHOME is a leukotoxin derivative of linoleic acid diol that has been reported to be toxic in human's tissue preparations, and is produced by inflammatory leukocytes such as neutrophils and macrophages [28], [29]. Mitochondrial dysfunction, suppression of neutrophil respiratory burst activity, increased cell oxidative stress, vasodilation, and apoptosis are features of 9,10-DiHOME toxicity [29], [30]. Mitochondria are a cellular target of 9,10-DiHOME and other leukotoxin diols [31]. In cell culture studies, Sisemore et al. [32] showed that 9,10-DiHOME disrupts mitochondrial function by altering inner membrane integrity and increasing cytochrome C release. The enhanced post-exercise increase in lysolipids (1- and 2-oleoylglycerophosphoethanolamine) during the pistachio arm of the study may reflect 9,10-DiHOME toxic effects on membrane integrity. ’The adverse effect of pistachio ingestion on performance may be related in part to the linkage between raffinose release from the colon, increased 9,10 DiHOME generation, and altered mitochondrial function.

Urate, a product of nucleotide degradation, is a potent scavenger of reactive oxygen species (ROS). Upon reaction with hydroxyl radicals, urate can be oxidized to a number of oxidation products including allantoin, and this has been observed in human skeletal muscle during supramaximal intensity exercise [33]. Elevated urine allantoin levels have been reported after intensive exercise and are considered to be an oxidative stress biomarker [34]. In this study, plasma allantoin was 2.55-fold higher post-exercise in the pistachio versus no pistachio trial, and was highly correlated with plasma raffinose. Taken together, these data indicate that colon-derived raffinose when combined with intensive and prolonged exercise induced significant ROS that was scavenged by urate.

In a previous metabolomics-based study with mice, azelaic acid was found to be an indicator of toxin exposure [35]. Azelaic acid is a byproduct of lipid oxidation of unsaturated fatty acids, and may interact with PPARs in the regulation of inflammatory processes [36]. Azelaic acid can be considered as an early marker of lipoprotein oxidation and subclinical atherosclerosis [37]. The 1.64-fold higher levels of azelaic acid post-exercise in the pistachio trial and high correlation with plasma raffinose is another indicator that raffinose was related to elevated oxidative stress and perhaps leukotoxin effects from 9,10-DiHOME. F_2_-IsoP was slightly elevated in the pistachio compared to water trial at 21-h post-exercise, but the P-value was not significant after Bonferroni correction. The allantoin and azelaic acid data support elevated oxidative stress in the pistachio trial immediately and 1.5-h post 75-km cycling, but not 21-h post-exercise. Despite the mismatch in timing, these data taken together support elevated oxidative stress in the pistachio trial.

There was a tendency for blood glucose levels to be maintained during the pistachio trial compared to a slight decrease during the no-pistachio trial. The 85 g serving of pistachios given to the cyclists before and during 75-km cycling provided 23 g carbohydrate, but only 8 g of pistachio carbohydrate is in the form of sugars and starches. In most studies where carbohydrate supplementation has been found to have influences on performance and exercise-induced changes in stress hormone and inflammation measures, athletes ingest 40–60 g carbohydrate (sugars) per hour of exercise [4], [5]. Thus the 8 g dose of sugars and starches provided in this study during the pistachio trial was too small to influence performance or any of the outcome measures, and this interpretation is supported by the lack of trial differences for the respiratory exchange ratio. The pistachio trial was related to elevations in several metabolites related to amino acid metabolism (leucine, indoleacetate, 2-aminooctanoate, gamma-glutamylleucine, gamma-glutamyltyrosine, phenylacetate, isobutyrylcarnitine) and fatty acid metabolism (2-hydroxydecanoate), and may have reflected the ingestion of pistachio nuts before and during exercise. Except for 2-hydroxydecanoate, trials differences were small and unlikely to have influenced high intensity cycling performance.

To summarize, in this metabolomics-based study we report an entirely novel finding that gut-derived raffinose, sucrose, and *myo*-inositol were present in the circulation of endurance athletes following the combination of 2-weeks pistachio nut ingestion and prolonged and intensive exertion. Pistachio ingestion was related to impaired 75-km cycling performance in overnight fasted cyclists, and could be due to raffinose associated increases in 9,10-DiHOME, a leukotoxin that may negatively impact mitochondrial function. No measure of mitochondrial function was measured in this study, and additional research is needed to test this hypothesis more carefully. Plasma raffinose following pistachio ingestion and 75-km cycling was also strongly correlated with oxidative stress indicators including allantoin and azelaic acid, but not the well-established lipid peroxidation biomarker, F_2_-isoprostanes, or cytokine inflammatory biomarkers. Together, these data support the value of utilizing metabolomics-based procedures in sports nutrition studies, and call into question the dietary practice of ingesting foods high in raffinose prior to long duration, intense exercise.

## Supporting Information

Checklist S1 **CONSORT Checklist.**
(DOC)

File S1 **Figures S1 to S19 depict box plots for the 19 metabolites in** **Table 5** **with significant treatment (pistachio, water) x time (5 points) interaction effects.**
(XLSX)

Protocol S1 **Trial Protocol.**
(PDF)

Table S1 **Nutrient profile of the 3 oz. (85 g) daily serving size of pistachio nuts used in this study.**
(XLSX)

Table S2 **Metabolomics analysis for detectable compounds of known identity.**
(XLSX)
