# Supplementary material for: Influence of Pistachios on Performance and Exercise-Induced Inflammation, Oxidative Stress, Immune Dysfunction, and Metabolite Shifts in Cyclists: A Randomized, Crossover Trial
Source: PLoS One. 2014 Nov 19;9(11):e113725. doi: 10.1371/journal.pone.0113725 (PMC4237504; doi:10.1371/journal.pone.0113725)
Supplement: Protocol S1 — Trial Protocol. (PDF) [file pone.0113725.s003.pdf]

For the best experience, open this PDF portfolio in Acrobat X or Adobe Reader X, or later.

Get Adobe Reader Now!
